# A randomized assessors-blind clinical trial to evaluate the safety and the efficacy of albendazole alone and in combination with mebendazole or pyrantel for the treatment of *Trichuris trichiura* infection in school-aged children in Lambaréné and surroundings

**DOI:** 10.1128/aac.01211-23

**Published:** 2024-04-02

**Authors:** Paul Alvyn Nguema Moure, Moustapha Nzamba Maloum, Gédéon Prince Manouana, Roméo-Aimé Laclong Lontchi, Mirabeau Mbong Ngwese, Jean Ronald Edoa, Jeannot Fréjus Zinsou, Brice Meulah, Saidou Mahmoudou, Elsy Mirna N'noh Dansou, Yabo Josiane Honkpehedji, Bayode Romeo Adegbite, Selidji Todagbe Agnandji, Michael Ramharter, Bertrand Lell, Steffen Borrmann, Peter G. Kremsner, Jean Claude Dejon-Agobé, Ayôla Akim Adegnika

**Affiliations:** 1 Centre de Recherches Médicales de Lambaréné, Lambaréné, Gabon; 2 Ecole Doctorale Régionale d’Afrique Centrale en Infectiologie Tropicale de Franceville, Franceville, Gabon; 3 Institute of Tropical Medicine, Universitätsklinikum Tübingen, Universität Tübingen, Tübingen, Germany; 4 Department of Parasitology, Leiden University Medical Center, Leiden, the Netherlands; 5 German Center for Infection Research (DZIF), Tübingen, Germany; 6 Department of Tropical Medicine, Bernhard Nocht Institute for Tropical Medicine & I. Dep. of Medicine, University Medical Center Hamburg-Eppendorf, Hamburg, Germany; 7 Division of Infectious Diseases and Tropical Medicine, Department of Medicine 1, Medical University of Vienna, Vienna, Austria; The Children's Hospital of Philadelphia, Philadelphia, Pennsylvania, USA

**Keywords:** *Trichuris trichiura*, coinfection, benzimidazole combination, albendazole, mebendazole, pyrantel, efficacy, safety, trichuriasis

## Abstract

Helminthiasis remains a public health issue in endemic areas. Various drugs have been proposed to improve efficacy against helminths. The study aimed to assess the safety and efficacy of three different anthelmintic combinations to treat *Trichuris trichiura* infections. We conducted a randomized assessors-blind clinical trial involving children aged 2–17 years with *T. trichiura*. Participants were randomly assigned to one of three treatment arms. On the first and third days, all participants got albendazole 400 mg, and on the second day, albendazole (arm A), mebendazole 500 mg (arm B), or pyrantel 125 mg/kg (arm C). We assessed treatment efficacy using the cure rate (CR) and egg reduction rate (ERR) at 3 and 6 weeks post-treatment. At 3 weeks post-treatment, ERR and CR were highest in study arm A [ERR = 94%, 95% confidence interval (CI): 92–95; CR = 71%; 95% CI: 58–81] compared to the B and C arms. Decrease in ERR was significant only for arm B versus arm A (*P*-value <0.001); decrease in ERR was significant for arms B and C (*P*-value <0.001). No statistical difference was observed in CR when comparing arms A and B (*P*-value =1.00) and C (*P*-value =0.27). At 6 weeks, a decrease in ERR was observed in three arms, significant only for arm C, 81% (95% CI: 78–83). A significant increase in egg counts was observed between 3 and 6 weeks post-treatment. All treatments were safe with mild adverse events. Albendazole 400 mg/day (arm A) showed the highest efficacy against trichuriasis. Nonetheless, this treatment regimen was able to cure half of the treated individuals highlighting concerns about controlling the transmission of *T. trichiura*.

**CLINICAL TRIAL:**

Registered at ClinicalTrials.gov (NCT04326868).

## INTRODUCTION

Soil-transmitted helminth (STH) infections remain a public health problem, affecting more than 1.5 billion people worldwide (1). They are prevalent in tropical regions, affecting mostly preschool-aged children (PSAC) and school-aged children (SAC) due to their play habits (2). Indeed, the 2020 World Health Organization (WHO) report indicates that over 267 million PSAC and over 568 million SAC live in the areas where these parasites are intensively transmitted (1). In addition, the poorest and most deprived communities due to the scarcity of drinkable water and adequate sanitation mainly contribute to the burden of the disease (1, 3). These data suggest that STH infections have a high burden among young children in African countries.

Humans are infected by STH as a result of contact with infesting forms of the worm. The most prevalent pathogenic STH species in humans are *Ascaris lumbricoides*, *Trichuris trichiura*, and hookworm (*Necator americanus* and *Ancylostoma duodenale*) (1, 4). The infections with these parasites are very often asymptomatic. However, for the severe form of the disease, clinical symptoms usually include discomfort, fatigue, diarrhea and abdominal pain, anemia, growth impairment, and cognitive and educational deficits (3). In areas where elimination of the disease is not yet possible, the WHO recommends the control of the disease morbidity in the communities through mass drug administration (MDA) campaigns of anthelminthic drugs to the population at-risk and improving environmental conditions as well as improvement of the water, sanitation, and hygiene (WASH) program. The aim of those programs is to reduce the prevalence and control the transmission of those diseases. Thus, the application of these preventive measures would be beneficial in fighting against this type of infection.

Large-scale administration of anthelmintic drugs is one of the strategies often applied in exposed communities to fight against STH infections. Benzimidazoles are drugs currently used for the treatment of STH infections. Nowadays, only four drugs are recommended by the WHO for the treatment of STH infections: mebendazole (MBZ), albendazole (ABZ), levamisole, and pyrantel (PYR) (5). For MDA of anthelminthic in the population at risk for STH, the WHO recommends a single-dose administration of ABZ 400 mg or MBZ 500 mg (1). Although these drugs have been shown to have good efficacy for the treatment of *A. lumbricoides* and hookworm infection (6), a concern remains about their capacity to totally clear the infection and control the transmission of the disease in endemic areas, particularly for *T. trichiura* infection. Indeed, a 95% and 90% reduction in egg load following treatment using ABZ is considered as satisfactory for *A. lumbricoides* and hookworm infections, respectively (7); the reduction in egg load by half for the same drug in the treatment of trichuriasis is nowadays considered as satisfactory (7), calling for a search for a more effective treatment or drug combination.

Egg reduction rate (ERR) and cure rate (CR) are two indicators recommended by the WHO to assess the efficacy of anthelminthic drugs. The ERR is considered the best indicator to assess the efficacy of drugs against human soil-transmitted helminth infections, and a high ERR and/or CR is often reported for ABZ and MBZ for the treatment of ascariasis and hookworm infection (8, 9). On the contrary, studies usually report for the same drug regimen a low ERR and/or CR of those drugs for *T. trichiura* (10, 11), making it difficult to manage their co-infection. Since STH species co-infection is common in endemic areas, there is a necessity to have effective treatment for all three main STH infections. In order to have such treatment, a repeated administration over consecutive days of the available drugs for the treatment of STH has been suggested, particularly to improve their efficacy for the treatment of trichuriasis. In that way, a better efficacy of two or three doses of ABZ administered on consecutive days compared to a single dose has been obviously reported for *A. lumbricoides* and hookworm species, but that efficacy remains lowest for *T. trichiura* (4, 12). The question could be whether the alternative administration of different available benzimidazoles including ABZ and MBZ can improve their efficacy in the treatment of trichuriasis. In the same vein, it could be interesting to know if the alternative administration of benzimidazoles with other anthelmintic such as pyronaridine derivatives can improve the efficacy of the treatment of trichuriasis while remaining highly effective for other STH infections. The objective of the present study was, therefore, to assess the efficacy of the combination of ABZ with MBZ or PYR for trichuriasis given alternately over 3 days apart to PSAC and SAC living in endemic areas of Gabon.

## MATERIALS AND METHODS

### Study design

This study was designed as a randomized, controlled, assessors-blind clinical trial to compare the efficacy and safety of different benzimidazole combinations administered on 3 consecutive days. Treated participants were seen 3 and 6 weeks post-treatment for stool sample collection to assess the efficacy of the drug combinations.

### Study populations

Participants aged 2–17 years, positive for *T. trichiura* infection, living in Lambaréné and surrounding areas, and without known chronic diseases and no history of anthelminthic treatment over the past 3 months were eligible to take part in the study.

### Study site and study areas

**Fig 1:**
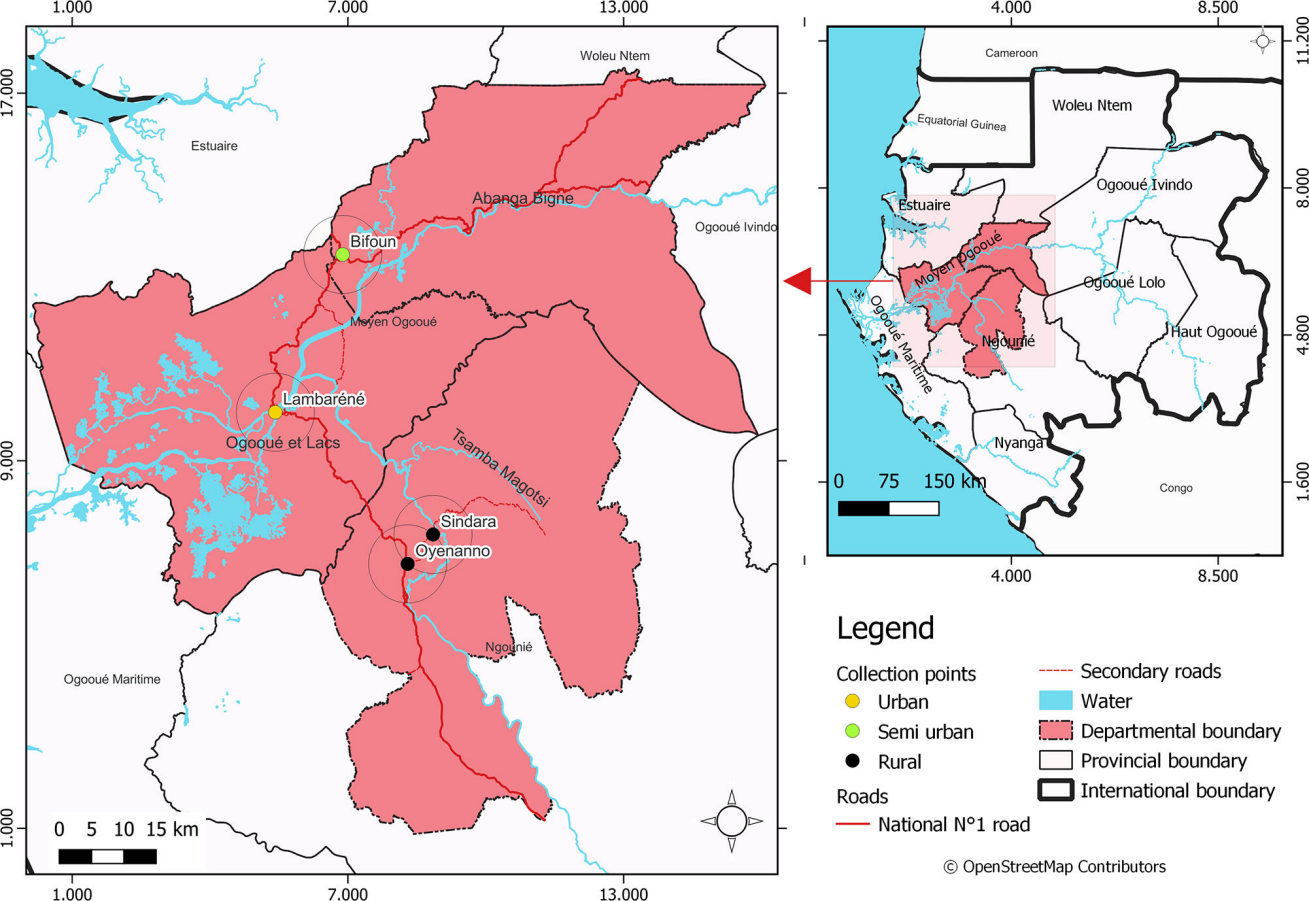
Map showing the study area. Right panel: provinces of Gabon with the study area highlighted in pink. Left panel: the departments where the study was conducted. ©OpenStreetMap contributors.

The study took place at the Centre de Recherches Médicales de Lambaréné (CERMEL) (13) and was conducted in the department of the Ogooué-et-des-Lacs of the Moyen-Ogooué province and the department of Tsamba-Magotsi in the Ngounié province (Fig. 1). In the Moyen-Ogooué province, the study was conducted in Lambaréné and surrounding areas. Lambaréné is the city capital of the province, which is known as a semi-urban area. Lambaréné is located 60 km south of the equator and is surrounded by a set of villages located along the N1 national road north and south of the main city. Lambaréné has about 45,000 inhabitants, while the surrounding rural areas have about 3,500 inhabitants (14). In the department of Tsamba-Magotsi, the study was conducted in Sindara and Oyenano, remote rural areas to Fougamou, the city capital of the province. The Tsamba-Magotsi department hosts 14,875 inhabitants. The study areas are known as endemic for STH infections, as well as other parasitic diseases including malaria, schistosomiasis, and filariasis (12, 15).

### Sample size calculation

Trichuriasis was our main STH infection of interest. A previous study conducted among children in the vicinity of Lambaréné reported 83% CR of three doses of ABZ for the treatment of trichuriasis (12). We hypothesize that the efficacy of the proposed treatment is better than what is observed for 400 mg of ABZ administered once a day for 3 consecutive days (reference arm). To be able to detect a minimum increase of 16% in the CR for trichuriasis, and considering a 95% confidence interval (α = 5%), a minimum of 51 participants per treatment arm was required, using the method described in 2004 by Humphry and collaborators (16).

### Randomization

Eligible participants were randomized in three different treatment arms: arm A (ABZ-ABZ-ABZ), arm B (ABZ-MLB-ABZ), and arm C (ABZ-PYR-ABZ). The randomization was carried out by a member of the research team not involved in other study procedures using a randomization list generated with R software version 4.1.0 (R Core Team, R Foundation for Statistical Computing, Vienna, Austria). The participants were assigned to one of the three treatment regimens on a 1:1:1 ratio.

### Study procedures

The recruitment of volunteers was conducted from November 2019 to August 2020. Due to the school closeout for COVID-19 restrictions, volunteers were seen at households to request their participation in the study. After the informed consent procedure of those willing to participate, sociodemographic information, vital signs, and anthropometric parameters were recorded using a standardized questionnaire. Weights were taken using a digital calibrated electronic balance with children wearing clothes and without shoes, while height was taken using a height pole mounted on the wall. In order to minimize intra-individual errors, all measurements were taken twice by different researchers, and the average value was calculated and used thereof. A screw-top stool container was given, and the participants were asked to provide fresh stool samples in the morning or the 7 following days of enrolment to the study. Stool samples were collected every morning and transported in an icebox directly to the CERMEL’s parasitology lab where the analysis was immediately performed. Laboratory technicians in charge of stool analysis were blinded to the assigned drug regimen. Participants positive for the presence of *T. trichiura* eggs were randomized in one of the three treatment arms. According to the treatment arm, drugs were prepared and put in a dark sealing bag by the member of the team responsible for randomization. The treatment was then scheduled, and the participant was seen at home where the treatment was administered by a dedicated team member not involved in other study procedures.

Before drug administration, participants were fed. After participants had taken drugs, they were observed for an hour by another study member who was blinded for the regimen allocation. After a time of direct observation by a member of the study team, participants were encouraged to contact the research team for any unforeseen event. The clinical investigator’s telephone contact was given to the participant to that end. In addition, participants were seen again 1 week later to further assess the occurrence of any treatment-related adverse events, notably digestive symptoms. Treated participants were visited for stool sample collection 3 (visit C1) and 6 (visit C2) weeks after treatment to assess the efficacy and safety of drug treatment. For stool samples provided at C1 and C2 visits, lab technicians had no information on the treatment received by the participant. All participants found positive for STH at the C2 visit received an additional dose of ABZ 400 mg as a national recommendation for STH infection treatment.

### Treatment arms

Three drugs were used as investigational products: albendazole (Lincoln Pharmaceutical Ltd) alone or alternatively with either mebendazole (Cadila Pharmaceutical Ltd) or pyrantel (Innothera Chouzy). Three arms of treatment were retained: arm A consisted of one tablet of ABZ 400 mg a day for 3 consecutive days, and for the other arms, one tablet of ABZ 400 mg on the first and third day, alternated on the second day with one tablet of either MBZ 500 mg (arm B) or PYR 125 mg (arm C) per body weight.

### Parasitological analysis

#### Kato-Katz

It is a laboratory method for preparing the human stool sample prior to searching for STH eggs under a microscope (17). Basically, the stool is homogenized from the container in which the sample was previously collected. A small quantity of stool is then taken with a spatula and sieved with metal mesh. Using the gabardine, 41.7 mg of sieved stool is collected and spread out on a slide and stained for 10 minutes using hydrophilic cellophane soaked in malachite-glycerol green prepared beforehand. The preparation is then examined using a low-power objective (×10) of a light microscope to identify and quantify helminth eggs. For each stool sample, two slides were prepared by two competent independent readers to quantify the egg load for STH. The readers were assigned to read the slide preparations independently. For positive samples, the arithmetic mean was calculated to determine the egg load. In the case of qualitative (positive *vs* negative) or quantitative (more than 10% difference in egg count) discrepancy between the two independent readers, a third reading was requested and was carried out by a third independent reader. In this case, the two closer results were considered for the calculation of egg count. The result was given in eggs per gram of stool.

### Statistical consideration

Data were collected using a patient report form and digitalized using the REDCap electronic data capture tool (18) hosted at CERMEL. Data analysis was performed using R software version 4.1.0 (R Core Team, R Foundation for Statistical Computing, Vienna, Austria). Age was used as a categorical variable. Age, height, and weight were used to calculate the height-for-age *Z*-score, weight-for-age *Z*-score (WAZ), and body mass index (BMI) using the WHO AnthroPlus software version 1.04 (WHO, Geneva, Switzerland) and categorized based on the WHO international reference values (available at http://www.who.int/growthref/tools/en/). Participants were considered infected with *T. trichiura* and other STH (*A. lumbricoides* and hookworms considered as a concomitant infection) if their stool samples were positive for the presence of at least one egg of the respective species. The intensity of infection was defined as described in Table S1. For stool samples collected 3 and 6 weeks post-treatment, the participant was considered cured if no egg was found in the sample for the respective species. Categorical variables were summarized by proportion and 95% confidence interval (95% CI) while continuous variables were summarized by mean and standard deviation (SD). The CR and ERR were used to assess the efficacy of different treatments and were calculated as described elsewhere (19) using the following formula:


$$CR=\frac{Number\;of\;subjects\;infected\;with\;STH\;who\;were\;cured\;}{Number\;of\;infected\;subjects\;who\;were\;treated}\times 100$$

$$ERR=\frac{Arithmetic\;mean\;EPG\;before\;treatment\;-\;Arithmetic\;\;mean\;after\;treatment}{Arithmetic\;\;mean\;EPG\;before\;treatment}\times 100$$

The binom.exact function of the Binom package in R was used to calculate the 95% CI of the CR and ERR, respectively. The difference in CR and ERR was determined under the assumption that no overlapping confidence intervals indicate statistical significance. In addition, the Chi-squared test was used to compare the ERR and CR between study arms and time points. The Kruskal–Wallis test was used to compare egg count between the study time points. The significance of all statistical tests performed was set at *P*-value <0.05. The number of adverse events (AEs) was summarized per arm to compare the safety of different treatment arms. The grading scale of the AEs was defined using the approach of the Food and Drug Administration in their guidance for the toxicity grading scale for volunteers enrolled in the preventive vaccine clinical trial (http://www.fda.gov/media/73679/download). Grade 1 (mild) was considered if the AE did not interfere with the participant’s activities, grade 2 (moderate) if some interferences with the participant’s activity were noted or medication was required, and grade 3 (severe) if the AE prevented the participant’s daily activity.

## RESULTS

### Study participant flow chart

**Fig 2:**
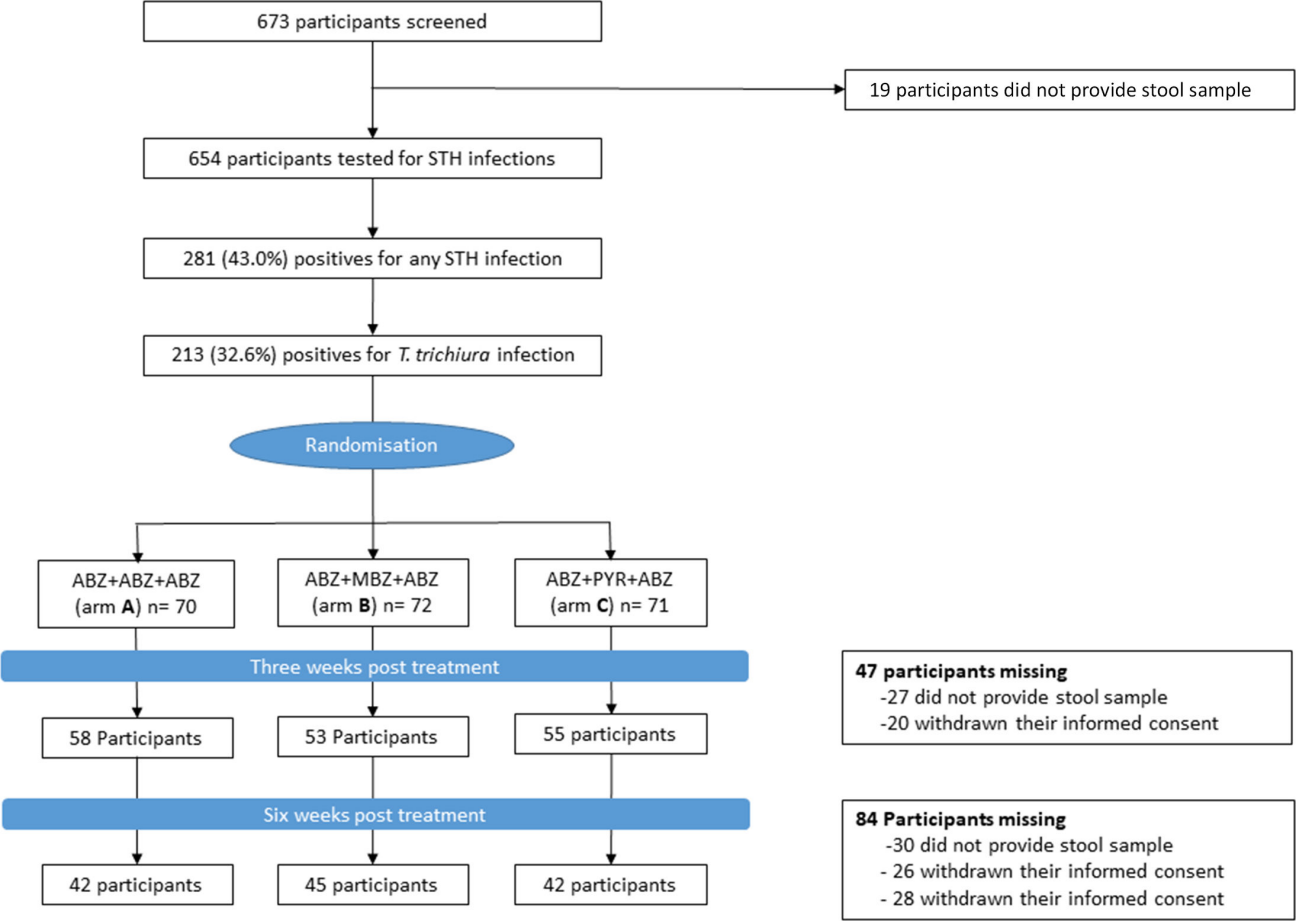
Study flow chart.

As described in Fig. 2, a total of 673 volunteers were invited to take part in the study, and only 654 of them provided stool samples for STH infection screening. After stool analysis, the 213 (32.6%) volunteers found positive for *T. trichiura* infection were randomized in one of the three study arms, giving the following number per arm of 70, 72, and 71 participants included in study arms A, B, and C, respectively. All randomized study participants were treated with respect to their study arm. At visit C1, 58, 53, and 55 participants in study arms A, B, and C, respectively, provided stool samples, while at visit C2, 42, 45, and 42 participants in each study arm provided stool samples, respectively (Fig. 2).

### Study population characteristics

The 213 volunteers found positive for the presence of *T. trichiura*’s eggs in stool constitute our study population. As described in Table 1, the mean (SD) age of the study population was 7.9 (3.5) years with a 1.0 female-to-male sex ratio. The study participants came mainly from rural areas (96%) while only eight (4%) participants came from Lambaréné, the semi-urban area. Age, sex, and locality were evenly distributed between the three study arms, while the BMI categories were similar between study groups A and C, but slightly different for study group B with the lowest proportion of participants overweight and obese and the highest proportion of those with normal weight or underweight (Table 1).

**TABLE 1:** Baseline characteristics of the 213 study participants

	Study population		Arm A: ABZ-ABZ-ABZ		Arm B: ABZ-MBZ-ABZ		Arm C: ABZ-PYR-ABZ	
	*n*	%	*n*	%	*n*	%	*n*	%
Total	213	–^*a*^	70	32.8	72	33.8	71	33.3
Sex								
Female	109	51.1	35	32.1	38	34.8	36	33.0
Male	104	48.8	35	33.6	34	32.6	35	33.6
Sex ratio (F/M)	1.0	–	1.0	–	0.9	–	1.0	–
Mean age (SD)	7.9 (3.5)	**–**	8.1 (3.7)	**–**	7.8 (3.3)	**–**	7.7 (3.6)	**–**
Age arm								
2–5	65	30.5	19	29.2	21	32.3	25	38.4
6–10	90	42.2	29	32.2	33	36.6	28	31.1
11–17	58	27.2	22	37.9	18	31.0	18	31.0
Body mass index								
Underweight	47	22.1	15	21.4	18	25.0	14	19.7
Normal	103	48.4	31	44.3	39	54.2	33	46.5
Overweight	32	15.0	13	18.6	9	12.5	10	14.1
Obese	31	14.5	11	15.7	6	8.3	14	19.7
Locality								
Lambaréné	8	3.7	03	37.5	2	25.0	3	37.5
Surroundings south of Lambaréné	109	51.1	33	30.2	36	33.0	40	36.6
Surroundings north of Lambaréné	96	45.0	34	35.4	34	35.4	28	29.1
*T. trichiura*	213	75.8	69	32.3	73	34.2	71	33.3
Light	176	82.6	55	31.2	64	36.6	57	32.3
Moderate	36	16.9	15	41.6	08	22.2	13	36.1
High	1	0.3	–	–	–	–	1	100.0

^*a*^ –, percentage calculation is not applicable.

### Distribution of STHs in the study population

Of the 213 participants positive for *T. trichiura* and included in the study, 176 (83%) presented light infection while the remaining 36 (17%) presented moderate infection (Table 2). Only one participant presented a heavy infection. Of the included participants, 96 (45%) and 39 (18%) were concomitantly positive for *A. lumbricoides* and hookworm, respectively. Considering the distribution per study arms, light trichuriasis represented 79%, 89%, and 80% in study arms A, B, and C, respectively, while the only case of heavy infection was included in study arm C. Co-infections with *A. lumbricoides* and hookworm were approximately similarly distributed between the study arms as presented in Table 2.

**TABLE 2:** Soil-transmitted helminth distribution in the study population and per arm of treatment at baseline^*a*^

	Study population		Arm A: ABZ-ABZ-ABZ		Arm B: ABZ-MBZ-ABZ		Arm C: ABZ-PYR-ABZ	
	*n*	%	*n*	%	*n*	%	*n*	%
*Trichuris trichiura*	213	100.0	70	100.0	72	100.0	71	100.0
Light	176	82.6	55	78.6	64	88.9	57	80.3
Moderate	36	16.9	15	21.4	8	11.1	13	18.3
High	1	0.3	0	–^*b*^	0	–	1	1.4
In co-infection with *A. lumbricoides*	96	45.0	30	42.9	36	50.0	30	42.2
Light	42	43.7	13	43.3	17	47.2	12	40.0
Moderate	39	40.6	10	33.3	14	38.9	15	50.0
High	15	15.6	7	23.3	5	13.9	3	10.0
In co-infection with hookworms	39	18.3	14	20.0	12	16.7	16	22.5
Light	37	94.8	12	85.7	11	91.7	15	100.0
Moderate	2	5.4	1	7.1	1	8.3	0	–
High	0	–	0	–	0	–	0	–

^*a*^ The classification of each parasite infection was done following the classification from WHO.

^*b*^ –, percentage calculation is not applicable.

### CR per treatment arm

As presented in Table 3, the CR at 3 weeks post-treatment was 71% (95% CI: 58–81), 62% (95% CI: 49–74), and 56% (95% CI: 43–69) in study arms A, B, and C, respectively. As compared to study arm A, no statistical difference was observed with study arm B (*P*-value =0.45) and study arm C (*P*-value =0.23), respectively. At 6 weeks post-treatment and as compared to the CR at 3 weeks post-treatment, a not statistically significant decrease in the CR was observed in study arm A (59%, 95% CI: 44–73), B (58%, 95% CI: 43–71), and C (45%, 95% CI: 31–60), respectively. As compared to study arm A, no difference was observed in the CR for study arm B (*P*-value =1) and study arm C (*P*-value =0.27), respectively. Taking into account the intensity of trichuriasis and co-infection with ascariasis and/or hookworm infection, a non-statistical decrease was observed either at 3 or 6 weeks post-treatment in CR when comparing study arm A with study arms B and C, respectively, except for those not co-infected. Indeed, the decrease in the CR observed at 3 weeks post-treatment in study arm C (53%; 95% CI: 36–70) when compared with study arm A (78%, 95% CI: 61–89) was statistically significant (*P*-value <0.001).

**TABLE 3:** CR distribution among study population for *T. trichiura*, at 3 and 6 weeks post-treatment^*b*^

	Study arm A: ABZ-ABZ-ABZ				Study arm B: ABZ-MBZ-ABZ					Study arm C: ABZ-PYR-ABZ				
	*N*	*n*	CR (%)	95% CI (CR)	*N*	*n*	CR (%)	95% CI (CR)	*P*-value	*N*	*n*	CR (%)	95% CI (CR)	*P*-value
3 post-treatment														
Trichuriasis														
Overall population	58	41	70.9	57.9–80.8	53	33	62.2	48.8–74.0	0.45	55	32	56.3	43.2–68.6	0.23
By intensity														
Light	46	33	71.7	57.4–82.6	45	30	66.6	52.0–78.6	0.76	44	24	54.4	40.0–68.2	0.14
Moderate	12	8	66.6	39.0–86.1	8	3	37.5	13.6–69.4	0.40	11	7	63.6	35.3–84.8	1.00
In co-infection^*a*^														
Yes	26	16	61.5	42.5–77.5	28	16	57.1	39.0–73.4	0.95	27	16	59.2	40.7–75.4	1.00
No	32	25	78.1	61.2–88.9	25	17	68	48.4–82.7	0.57	28	15	53.5	35.8–70.4	<0.001
6 weeks post-treatment														
Trichuriasis														
Overall population	42	25	59.5	44.4–72.9	45	26	57.7	43.3–71.0	1.00	42	19	45.2	31.2–60.0	0.27
By intensity														
Light	34	21	61.7	45.0–76.0	38	22	57.8	42.1–72.1	0.92	31	15	48.3	31.9–65.1	0.40
Moderate	8	4	50.0	21.5–78.4	7	4	57.1	25.0–84.1	1.00	11	4	36.6	15.1–64.6	0.90
In co-infection^*a*^														
Yes	24	13	54.1	35.0–72.1	24	13	54.1	35.0–72.1	1.00	24	9	37.5	21.1–57.2	0.38
No	18	12	66.6	43.7–83.7	21	13	61.9	40.8–79.2	1.00	18	10	55.5	33.7–75.4	0.73

^*a*^ Co-infection with *Ascaris lumbricoides* and/or hookworm.

^*b*^ *N*, number of participants infected with the respective STH species at baseline and seen at the control visit; *n*, number of children cured.

### ERR distribution

The ERR for trichuriasis of 94% (95% CI: 92–95), 87% (95% CI: 83–89), and 95% (95% CI: 93–96) was observed in study arms A, B, and C, respectively (Table 4). As compared to study arm A, the lower ERR observed with study arm B was statistically significant (*P*-value <0.001), while no difference was observed with study arm C (*P*-value =0.45). Taking into account the intensity of the disease, a statistically significant decrease was observed among participants with moderate intensity when comparing study arm B and study arm A (94% vs 98%, *P*-value <0.001), while on the contrary, a statistically significant increase in the ERR was observed when comparing study arm C with study arm A (99% vs 98%, *P*-value <0.001). No difference in the ERR was observed for those with light infection and similarly when considering co-infection with *A. lumbricoides* and hookworm. At 6 weeks post-treatment, the ERR was 92% (95% CI: 90–94), 77% (95% CI: 79–84), and 81% (95% CI: 78–83) in study arms A, B, and C, respectively. As compared to 3 weeks post-treatment, the decrease observed was statistically significant only in study arm C. As compared to study arm A, the decrease observed in ERR was statistically significant for study arms B (*P*-value <0.001) and C (*P*-value <0.001), respectively. A similar decrease was observed when considering the intensity of trichuriasis (*P*-value <0.01) and co-infection with *Ascaris* and/or hookworm (*P*-value <0.001).

**TABLE 4:** ERR following treatment of trichuriasis infections at 3 and 6 weeks post-treatment^*b*^

	Study arm A: ABZ-ABZ-ABZ				Study arm B: ABZ-MBZ-ABZ					Study arm C: ABZ-PYR-ABZ				
	*M*	*m*	ERR (%)	95% CI (ERR)	*M*	*m*	ERR (%)	95% CI (ERR)	*P*-value	*M*	*m*	ERR (%)	95% CI (%)	*P*-value
3 weeks post-treatment														
*Trichuris trichiura*														
Overall	733	46	93.7	91.7–95.2	593	77	87.0	84.0–89.4	<0.001	820	43	94.7	93.0–96.0	0.44
By intensity														
Light	265	48	81.8	76.8–86.0	248	62	73.7	67.9–78.8	0.03	226	49	78.3	72.4–83.1	0.38
Moderate	2,413	42	98.2	97.6–98.7	2,812	171	93.9	92.9–94.7	<0.001	2,382	14	99.4	99.0–99.6	<0.001
In co-infection^*a*^														
Yes	1,101	64	94.1	92.6–95.4	916	123	86.5	84.2–88.6	<0.001	1,035	39	96.2	94.8–97.2	0.03
No	429	32	92.5	89.6–94.6	217	24	88.9	84.0–92.4	0.16	613	46	92.4	90.1–94.3	1.0
6 weeks post-treatment														
*Trichuris trichiura*														
Overall	725	59	91.8	89.6–93.6	688	156	77.3	79.3–84.4	<0.001	880	170	80.6	77.9–83.1	<0.001
By intensity														
Light	234	44	81.1	75.7–85.6	244	78	68.0	61.9–73.5	0.01	250	153	38.8	32.9–44.9	<0.001
Moderate	2,815	121	95.7	94.8–96.3	2,797	523	81.3	79.8–82.7	<0.001	2,500	292	88.3	87.0–89.5	<0.001
In co-infection^*a*^														
Yes	3,065	138	95.4	94.7–96.1	1,113	254	77.17	74.1–79.5	<0.001	770	249	67.6	64.2–70.8	<0.001
No	1,065	0	100.0	99.6–100	225	48	78.6	72.8–83.5	<0.001	797	116	85.4	82.8–87.7	<0.001

^*a*^ Co-infection with *Ascaris lumbricoides* and/or hookworm.

^*b*^ *M*, arithmetic mean of egg count at baseline; *m*, arithmetic mean of egg count at 3 or 6 weeks post-treatment.

### *Trichuris trichiura* egg dynamic

**Fig 3:**
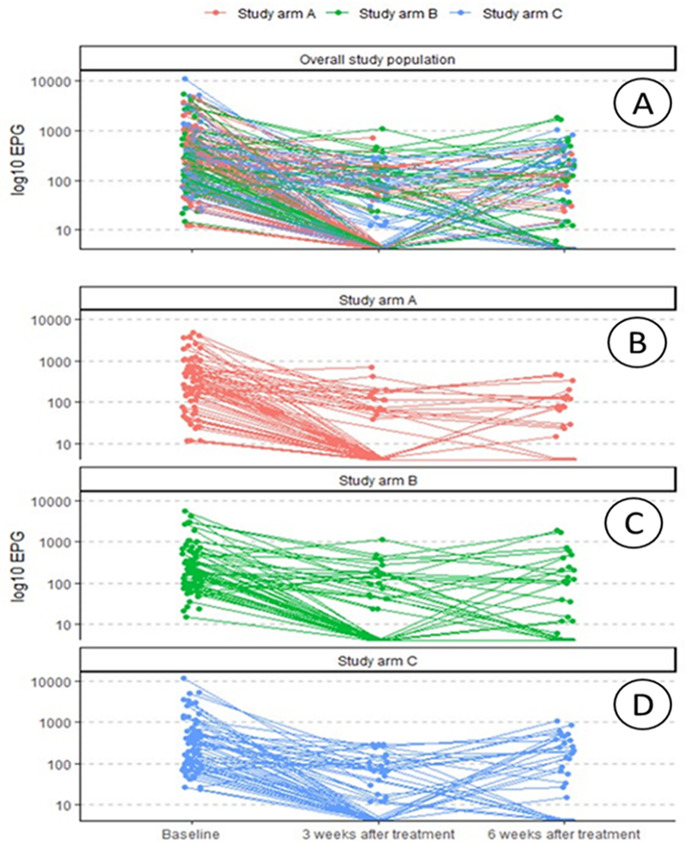
Spaghetti plot distribution of egg loads from *T. trichiura* per time point and study arm. Panel (A): overall study population. Panel (B): study arm A (treatment regimen: albendazole + albendazole + albendazole). Panel (C): study arm B (treatment regimen: albendazole + mebendazole + albendazole). Panel (D): study arm C (treatment regimen: albendazole + pyrantel + albendazole).

Figure 3 depicts the dynamic of *T. trichiura* egg count observed from baseline to 3 and 6 weeks post-treatment and per study arm. The overall trend shows a decrease in egg count intensity from baseline to 3 weeks and an increase in egg counts from 3 to 6 weeks (Fig. 3A). Considering the study arms (Fig. 3B), we observed a statistically significant (*P*-value <0.001) reduction in egg load per gram in the three study arms at 3 weeks, as compared to baseline. At 6 weeks as compared to 3 weeks, a statistically significant increase in egg load was observed in all study groups (*P*-value =0.001). When comparing egg count between baseline and 6 weeks post-treatment, no statistically significant difference was found in the three study groups (*P*-value =0.08).

### Safety of different treatments

**Fig 4:**
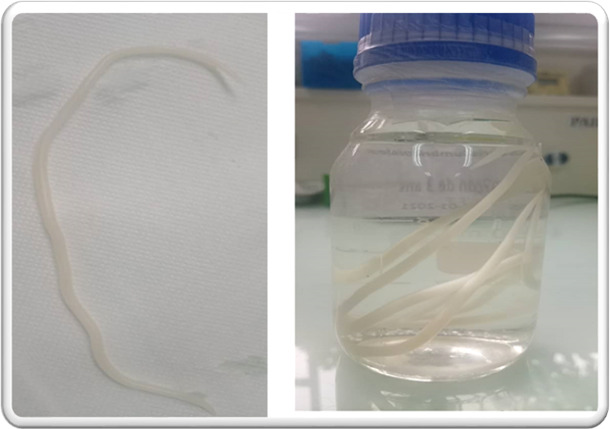
Adult *Ascaris lumbricoides* worm (left panel) vomited on the third day of treatment by a 3-year-old study participant. The right panel shows the worm preserved in a box containing formalin. (Images are from the CERMEL parasitology laboratory, January 2021.)

Table 5 reports the AEs recorded in the frame of the present study. Basically, 19 (8.9%) of the study population experienced at least one AE which included eight cases of pruritus, eight cases of abdominal pain (where three were followed by expelled worm in stool), three cases of throat discomfort (with expelled worm by mouth), and 12 cases of fever all reported from day 2 of the treatment to day 4. Indeed, pruritus was reported mainly on day 4 while abdominal pain was reported from day 2 to day 4. All cases of fever were found positive for *Plasmodium* parasites and were considered as malaria attacks, with three attacks appearing during the treatment phase and the other nine during the follow-up phase. No difference in the distribution of malaria attacks was observed between the study arms (*P*-value =0.32). All reported AEs were grade 1 and were considered as related to the treatments. In addition, the three participants who reported abdominal pain also reported tingling in the throat and expelled *Ascaris lumbricoides* worms (Fig. 4) either by the mouth or through feces from day 2 (two participants) to day 4 of the study course. Two other study participants expelled worms through feces. All AEs were well balanced between the study arms. Indeed, no statistically significant difference was observed in the distribution of AEs between study arms (*P*-value = 0.44).

**TABLE 5:** Proportion of the AEs and concomitant malaria infection observed during the treatment

Adverse events	General population		Study arm A: ABZ-ABZ-ABZ		Study arm B: ABZ-MBZ-ABZ		Study arm C: ABZ-PYR-ABZ		p-value
	n	%	n	%	n	%	n	%	
Study population	213	-^*a*^	70	-	72	-	71	-	-
Overall number of AEs	19	8.9	7	10.00	4	5.5	8	11.3	0.44
Pruritis	8	42.1	3	42.9	1	25.0	4	50.0	0.26
Grade 1	8	100.0	3	100.0	1	100.0	4	100.0	
Grade 2	0	-	0	-	0	-	0	-	
Grade 3	0	-	0	-	0	-	0	-	
Abdominal pain	3	15.8	1	14.3	1	25.0	1	12.5	1.00
Grade 1	3	100.0	1	100.0	1	100.0	1	100.0	
Grade 2	0	-	0	-	0	-	0	-	
Grade 3	0	-	0	-	0	-	0	-	
Abdominal pain with expel worm in stool	5	26.3	2	28.6	1	25.0	2	25.0	0.74
Grade 1	5	100.0	2	100.0	1	100.0	2	100.0	
Grade 2	0	-	0	-	0	-	0	-	
Grade 3	0	-	0	-	0	-	0	-	
Throat discomfort with expel worm by mouth	3	15.8	1	14.3	1	25.0	1	12.5	1.0
Grade 1	3	100.0	1	100.0	1	100.0	1	100.0	
Grade 2	0	-	0	-	0	-	0	-	
Grade 3	0	-	0	-	0	-	0	-	
Uncomplicated malaria	12	5.6	2	2.9	5	6.9	5	7.0	0.32

^*a*^ –, percentage calculation is not applicable.

## DISCUSSION

In the present study, we aimed to assess the efficacy of two proposed anthelminthic combinations for the treatment of trichuriasis, as compared to 400 mg of ABZ a day for 3 consecutive days. One dose of ABZ over a 3-day regimen was previously assessed in the local population showing a better efficacy, as compared to one dose of ABZ 400 mg alone (12), and is already implemented in the country for the treatment of STH infection. Our results confirm the good efficacy of that regimen for the treatment of trichuriasis but reveal a reduced efficacy of the two proposed anthelminthic combinations as compared to the reference. Moreover, we observed a reduction in the efficacy in time of the proposed combinations contrary to ABZ over 3 days, which remained stable. A significant increase in parasite egg count at 6 weeks post-treatment was thus noticed after a first decrease observed at 3 weeks. The two new combinations are proposed based on their mechanism of action; ABZ and MBZ deprive the worm of sugar, while PYR is known to paralyze the worm that “loses its grip” on the intestinal wall and then passes out of the digestive system by natural process (20). By combining ABZ with MBZ or PYR, we expected to find a regimen with better efficacy for the treatment of *T. trichiura* infection.

We used the ERR as an indicator of efficacy for the treatment of *T. trichiura* infection. Our results showed a high ERR for the three proposed treatment regimens (ERR ≥ 87%), all considered satisfactory according to WHO’s criteria (21, 22). The result for ABZ we reported over 3 days is in line with the 91% (95% CI: 83–100) ERR previously reported in the same population a decade ago (12), but different from those obtained in Ivory Coast by Patel and colleagues in 2020 where an increased dose from 400 to 600 and 800 mg did not improve the efficacy of ABZ for the treatment of trichuriasis, with the conclusion that albendazole is not an effective treatment for *T. trichiura* even at the highest doses administered (23). The difference observed could be explained by the duration of the treatment, as ABZ was administered once instead of 3 days as we did. Of the two proposed combinations, ABZ in combination with PYR has shown similar efficacy as repeated administration of ABZ over 3 days, while ABZ in combination with MBZ has shown a reduced efficacy as the repeated administration of ABZ. If this was true at 3 weeks post-treatment, a different pattern was observed 6 weeks post-treatment where the two proposed combinations of anthelminthic become less effective than the repeated administration of ABZ only. This pattern would be exacerbated in case of light intensity of trichuriasis or in case of co-infection with other STH. This finding suggests that ABZ remains the best molecule for the treatment of trichuriasis, but there is a need to expose the parasite for a sufficient period (3 days and probably more) to be effective. In the two proposed combinations, ABZ was administered 2 days apart. Replacing ABZ on 1 day with other molecules did not improve the outcome of the treatment. Administration of 400 mg of ABZ for 3 consecutive days, therefore, remains the best combination, as compared to the two other combinations: ABZ+MBZ+ABZ and ABZ+PYR+ABZ. To reach 100% of the ERR using ABZ in the treatment of trichuriasis as was the case for ascariasis and hookworm infection in our cohort (Table S2), one could suggest increasing the dose of ABZ on at least 1 of the 3 treatment days or administrating 400 mg of ABZ for more than 3 days. Such treatment will be relevant for the control of both morbidity and transmission of the disease in a community.

To assess the drug efficacy for the treatment of STH infections, it is recommended to collect stool samples for the control 2 or 3 weeks post-treatment, first to standardize the procedure, but mainly to avoid the risk that eggs identified in a stool specimen during the control are from parasites that infected the individual after treatment (22). In the present study, in addition to 3 weeks (21 days) post-treatment, we also collected stool samples at 6 weeks (42 days) post-treatment. Regarding the egg dynamic after treatment, a significant increase was observed in egg production between week 3 and week 6 post-treatment, reaching an absence of difference in egg load as compared to before treatment. A similar result was obtained with ABZ in Malaysia by Tee et al. in 2022 in a similar study design, and the author suggested that the observed result may be due to a treatment failure or incomplete action of the ovicidal, larvicidal, or vermicidal effect of benzimidazole drugs against *T. trichiura* (24, 25). If we assume that the increase in the number of *T. trichiura* eggs observed on day 42 post-treatment in the study arm with the ABZ-PYR combination could be explained, for example, by the release of worms from “insufficient” pyrantel-induced paralysis, we agree with Tee and collaborators regarding the incomplete effect of ABZ and MBZ on *T. trichiura* worm (24). Indeed, it has been demonstrated that *T. trichiura* worms have the ability to embed themselves in the intestinal mucosa, which prevents them from the action of benzimidazole drugs, ABZ, for instance, and allows them to recover after treatment (24). This hypothesis sustains the idea of the time-dependent effect of ABZ in the treatment of trichuriasis. It is also known that *T. trichiura* is able to excrete the drug using transport mediated by glycoprotein P (24, 25), which could explain our results. However, the mechanism sustaining the egg dynamic over time following treatment of trichuriasis needs to be properly investigated, and treatment failure should be clearly differentiated from early reinfection.

The main objective of treatment is to cure people infected with a disease. In the case of STH infections, despite the efficacy of the drug, some individuals may remain infected, which could thus perpetuate the transmission of the disease in the community. Here, we used the CR to assess the capacity of the different proposed treatments to cure individuals with trichuriasis. Our results revealed that ABZ-MBZ-ABZ and ABZ-PYR-ABZ combinations have similar performance in terms of patient cured as repeated administration of ABZ over 3 days, with approximately one individual cured out of two treated, and irrespective of the intensity of trichuriasis or its co-infection with other STH. These results indicate that the proposed treatments are not able to control or interrupt the transmission of trichuriasis in our community, which, therefore, remains a concern. For efficient control of the transmission of *T. trichiura* infection, there is a necessity for the implementation of additional measures such as the WASH program and vaccine. Such a vaccine against hookworm is currently under assessment in our study population (26).

The safety of our proposed anthelminthic combinations for the treatment of trichuriasis was one of our main objectives. Basically, all treatment regimens were well tolerated and had similar safety. Indeed, the AEs reported in the three study arms were all of grade 1, with no difference between study arms in the term type of AE (Table 5). We mainly reported pruritus and abdominal pain as AEs, with some participants expelling *Ascaris lumbricoides* worms by mouth and in the feces. Those AEs occurred mainly at 24 and 48 hours from the first day of treatment. Although with different anthelminthic combinations, Knopp et al. in 2010 reported no difference in terms of AE distribution between regimen treatments for the treatment of trichuriasis: albendazole or mebendazole administered alone or in combination with ivermectin. However, the author reported more AEs than we did, which included mainly abdominal cramps, headache, nausea, diarrhea, and allergic reactions (27). The difference observed could be due to the molecule included in the combination: ivermectin for Knoop et al., instead of pyrantel. Using an analog of pyrantel in one of their combination, Speech and colleagues in 2015 reported on their side a higher number of abdominal cramps and headaches among participants who received albendazole plus oxantel pamoate than those who received albendazole alone or in combination with ivermectin, with all AEs mild (28). These results indicate at least the safety of anthelminthic drugs used in combination, with a limited number of AE types. In our study, no specific AEs of the proposed combination were observed.

Interestingly, we observed cases of malaria attack following treatment. Although the link with the administration of anthelminthic drugs could be difficult to establish, one can hypothesize that death of worms, including *T. trichiura*, *A. lumbricoides*, hookworm, and even *Strongyloides stercoralis* all sensible to the drugs even at different levels, would have lifted a potential protection of STH against the development of symptoms and in particular fever in malaria. Indeed, *A. lumbricoides,* for instance, was found to have a role in the establishment of malaria tolerance in Thai patients (29). This calls for further investigation into the role of helminth infections in malaria development.

The effect of inter-individual variances such as body weight known to potentially affect the drug bioavailability may be considered as a concern. This issue was not specifically addressed in our study but should be controlled through the randomization process. The mean *z*-scores (weight for age and height for age) between the study groups were comparable (Table S3). In addition to the randomization, all participants were fed evenly before the administration of the drug to reduce factors influencing drug bioavailability in our study population. Another limitation of the present study could be the high number of withdrawals observed over the study course, mainly due to the difficulty of some participants to provide stool samples when requested. Indeed, if the number of participants present at 3 weeks post-treatment was higher than the minimum number requested for the efficacy assessment at that time point and did not, therefore, affect the main objective of our study, the about 60% withdrawn at 6 weeks could have an impact on our conclusion on the efficacy at 6 weeks. However, the statistically significant difference observed particularly in the ERR at 6 weeks when comparing study arm A *vs* study arms B and C, respectively, could assume that the sample size we had at 6 weeks post-treatment is enough to answer our exploratory objective. Indeed, considering all missing participants, either positive or negative did not affect our conclusion on the CR (Table S4). In addition, we reported an increase in egg count from 3 to 6 weeks but did not assess the viability of those eggs at a 6-week time point. We cannot, therefore, confirm that eggs found at that time point come from live *T. trichiura* worms.

### Conclusion

The present study highlights the efficacy of a 3-day course of albendazole 400 mg in reducing egg counts during the treatment of trichuriasis. However, when albendazole is replaced with mebendazole or pyrantel on the second day of treatment, this efficacy is reduced. It is worth noting that the treatment regimen was only able to cure half of the individuals treated. This suggests that these regimens can significantly reduce morbidity issues associated with trichuriasis but may not be suitable for controlling the transmission of the disease in endemic areas. This underscores the importance of implementing the WASH program in such areas. Therefore, a new regimen and agent are needed for the control of trichuriasis. Furthermore, the observation of an increase in egg counts after an initial decrease in trichuriasis raises questions about the optimal timing for assessing drug efficacy in the treatment of trichuriasis. It also highlights the need to distinguish between reinfection and treatment failure in cases of trichuriasis.

## SUPPLEMENTAL MATERIAL

Supplemental tables aac.01211-23-s0001.docx Tables S1 to S4.

ASM does not own the copyrights to Supplemental Material that may be linked to, or accessed through, an article. The authors have granted ASM a non-exclusive, world-wide license to publish the Supplemental Material files. Please contact the corresponding author directly for reuse.
