# Supplementary material for: A randomized assessors-blind clinical trial to evaluate the safety and the efficacy of albendazole alone and in combination with mebendazole or pyrantel for the treatment of Trichuris trichiura infection in school-aged children in Lambaréné and surroundings
Source: Antimicrob Agents Chemother. 2024 Apr 2;68(5):e01211-23. doi: 10.1128/aac.01211-23 (PMC11064543; doi:10.1128/aac.01211-23)
Supplement: Supplemental tables — Tables S1 to S4. [file aac.01211-23-s0001.docx]

**Supplementary Table S1.** Classes of intensity for soil-transmitted helminth infections [1]

| Organism | Light-intensity infection (epg*) | Moderate-intensity infection (epg) | Heavy-intensity infection (epg) |
| --- | --- | --- | --- |
| *Ascaris lumbricoides* | 1 – 4999 | 5 000 – 49 999 | ≥ 50 000 |
| *Trichuris trichiura* | 1 – 999 | 1 000 – 9 999 | ≥ 10 000 |
| Hookworms | 1 – 1 999 | 2 000 – 3 999 | ≥ 4 000 |

*Egg per Gram

Reference:

1. WHO Expert Committee. Prevention and control of schistosomiasis and soil-transmitted helminthiasis. World Health Organ Tech Rep Ser. 2002;912:i-vi, 1-57, back cover. PMID: 12592987.

**Supplementary Table S2.** Egg reduction rate (ERR) following treatment of *A. lumbricoides*, and hookworm infection evaluated at three- and six-weeks post treatment

|  | **Study arm A:**  albendazole-albendazole-albendazole | | | | |  | **Study arm B:**  albendazole-mebendazole-albendazole | | | | |  | **Study arm C:**  albendazole-pyrantel-albendazole | | | | |
| --- | --- | --- | --- | --- | --- | --- | --- | --- | --- | --- | --- | --- | --- | --- | --- | --- | --- |
|  | **N** | **M** | **m** | **ERR (%)** | **95%CI (%)** |  | **N** | **M** | **m** | **ERR (%)** | **95%CI (%)** |  | **N** | **M** | **m** | **ERR (%)** | **95%CI (%)** |
| **Three weeks post treatment (C1 visit)** | | | | | | | | | | | | | | | | | |
| *A. lumbricoides* | 35 | 33737 | 14 | **99.9** | 99.9 – 100.0 |  | 33 | 37484 | 6 | **99.9** | 99.9 – 100.0 |  | 34 | 31122 | 0 | **100.0** | 99.9 – 100.0 |
| Hookworm | 15 | 486 | 0 | **100.0** | 99.2 – 100.0 |  | 11 | 482 | 0 | **100.0** | 99.2 – 100.0 |  | 12 | 111 | 6 | **94.6** | 88.6 – 98.0 |
| **Six weeks post treatment (C2 Visit)** | | | | | | | | | | | | | | | | | |
| *A. lumbricoides* | 29 | 28207 | 38 | **99.9** | 99.8 – 99.9 |  | 25 | 29532 | 42 | **99.9** | 99.8 – 99.9 |  | 33 | 29532 | 0 | **100.0** | 99.9 – 100.0 |
| Hookworm | 10 | 612 | 0 | **100.0** | 99.4 – 100.0 |  | 9 | 553 | 0 | **100.0** | 99.3 – 100.0 |  | 8 | 281 | 0 | **100.0** | 98.7 – 100.0 |

M: arithmetic mean at baseline; m: arithmetic mean; CI: confidence interval; ERR: egg reduction rate

**Supplementary Table S3.** Anthropometric and biologic parameters per treatment arm

|  | | Study arm A: ABZ-ABZ-ABZ | Study arm B: ABZ-MBZ-ABZ | Study arm C: ABZ-PYR-ABZ |
| --- | --- | --- | --- | --- |
|  |  | Mean (SD); [IC 95%] | Mean (SD) ; [IC 95%] | Mean (SD) ; [IC 95%] |
| BMI (SD) | | 24.67 (8.22); [21.71-26.62] | 22,65 (5.55) ; [21,33-23,96] | 24,29 (6.80) ; [22.69-25.88] |
| Z score Weight for age (WAZ) | | -0.88 (1.8); [-1.30- -0.45] | -1.12 (1.54) ; [-1.48- -0.75] | -1.36 (1.56) ; [-1.72- -0.99] |
| Z score Height for age (HAZ) | | -4.86 (3.11); [-5.60- -4,11] | -4.78 (2.37) ; [-5.34- -4.21] | -5.48 (2.56) ; [-6,08- -4.87] |
| Haemoglobin (HGB) | | 10.13 (1.77); [9.70-10.55] | 10.17 (1.71) ; [9.76-10.57] | 10.20 (1.75) ; [9,78-10,61] |
| Transaminases | |  |  |  |
|  | **Alanine Aminotransferase (ALTL)** | 16.25 (8.02); [14.33-18.16] | 16.16 (8.52) ; [14,15-18.16] | 16.61 (8.79) ; [14.56-18.69] |
|  | **Asparate aminotransferase (ASTL)** | 27.46 (8.30); [25.48-29,43] | 27.14 (8.15) ; [25,22-29,05] | 27.47 (8.22) ; [25.52-29.41] |

ABZ: Albendazole; MBZ: Mebendazole; PYR: Pyrantel

**Supplementary Table S4.** Cure rate calculated at three- and six-weeks post treatment, by considering missing participants either as positive or as negative for trichuriasis.

|  | | | Study arm A: ABZ-ABZ-ABZ | | | |  | Study arm B: ABZ-MBZ-ABZ | | | | |  | Study arm C: ABZ-PYR-ABZ | | | | |
| --- | --- | --- | --- | --- | --- | --- | --- | --- | --- | --- | --- | --- | --- | --- | --- | --- | --- | --- |
|  |  |  | N | n | CR(%) | 95%CI(CR) |  | N | n | CR(%) | 95%CI(CR) | *P-value* |  | N | n | CR(%) | 95%CI(CR) | *P-value* |
| Lost to follow up as negative for trichuriasis | | | | | | | | | | | | | | | | | | |
| Three weeks post treatment | | | | | | | | | | | | | | | | | | |
|  | | Overall population | 70 | 41 | 58.7 | 46.8 – 69.3 |  | 71 | 33 | 46.4 | 35.5 – 57.9 | 0.20 |  | 72 | 32 | 44.4 | 33.5 – 55.9 | 0.12 |
| Six weeks post treatment | | | | | | | | | | | | | | | | | | |
|  | Overall population | | 70 | 25 | 35.7 | 25.5 – 47.4 |  | 71 | 26 | 36.6 | 26.3 – 48.2 | 1.00 |  | 72 | 19 | 26.3 | 17.5 – 37.5 | 0.30 |
| Lost to follow up as positive for trichuriasis | | | | | | | | | | | | | | | | | | |
| Three weeks post treatment | | | | | | | | | | | | | | | | | | |
|  | | Overall population | 70 | 53 | 75.7 | 64.5 – 84.2 |  | 71 | 51 | 71.8 | 60.4 – 80.9 | 0.73 |  | 72 | 49 | 68.0 | 56.6 – 77.7 | 0.40 |
| Six weeks post treatment | | | | | | | | | | | | | | | | | | |
|  | Overall population | | 70 | 37 | 52.8 | 41.3 – 64.0 |  | 71 | 44 | 61.9 | 50.3 – 72.3 | 0.35 |  | 72 | 36 | 50.0 | 38.7 – 61.2 | 0.86 |
